# Rhythm and Melody Tasks for School-Aged Children With and Without Musical Training: Age-Equivalent Scores and Reliability

**DOI:** 10.3389/fpsyg.2018.00426

**Published:** 2018-04-05

**Authors:** Kierla Ireland, Averil Parker, Nicholas Foster, Virginia Penhune

**Affiliations:** ^1^Penhune Laboratory for Motor Learning and Neural Plasticity, Department of Psychology, Concordia University, Montreal, QC, Canada; ^2^International Laboratory for Brain, Music and Sound Research, Montreal, QC, Canada

**Keywords:** musical tasks, school-aged children, age-equivalent scores, discrimination, synchronization

## Abstract

Measuring musical abilities in childhood can be challenging. When music training and maturation occur simultaneously, it is difficult to separate the effects of specific experience from age-based changes in cognitive and motor abilities. The goal of this study was to develop age-equivalent scores for two measures of musical ability that could be reliably used with school-aged children (7–13) with and without musical training. The children's Rhythm Synchronization Task (c-RST) and the children's Melody Discrimination Task (c-MDT) were adapted from adult tasks developed and used in our laboratories. The c-RST is a motor task in which children listen and then try to synchronize their taps with the notes of a woodblock rhythm while it plays twice in a row. The c-MDT is a perceptual task in which the child listens to two melodies and decides if the second was the same or different. We administered these tasks to 213 children in music camps (musicians, *n* = 130) and science camps (non-musicians, *n* = 83). We also measured children's paced tapping, non-paced tapping, and phonemic discrimination as baseline motor and auditory abilities We estimated internal-consistency reliability for both tasks, and compared children's performance to results from studies with adults. As expected, musically trained children outperformed those without music lessons, scores decreased as difficulty increased, and older children performed the best. Using non-musicians as a reference group, we generated a set of age-based z-scores, and used them to predict task performance with additional years of training. Years of lessons significantly predicted performance on both tasks, over and above the effect of age. We also assessed the relation between musician's scores on music tasks, baseline tasks, auditory working memory, and non-verbal reasoning. Unexpectedly, musician children outperformed non-musicians in two of three baseline tasks. The c-RST and c-MDT fill an important need for researchers interested in evaluating the impact of musical training in longitudinal studies, those interested in comparing the efficacy of different training methods, and for those assessing the impact of training on non-musical cognitive abilities such as language processing.

## Introduction

Researchers, music teachers, and parents have a strong interest in understanding and assessing children's musical abilities. However, measuring these abilities in childhood can be a challenge because training and normal maturation occur simultaneously, making it difficult to disentangle the effects of music experience from cognitive and motor development (Galván, 2010; Corrigall and Schellenberg, 2015). This also makes comparisons with adult musicians problematic. Therefore, the goals of this study were to develop measures of musical ability that could be reliably used with school-aged children (7–13), and to generate a set of age-based scores for children with and without training. The resulting children's Rhythm Synchronization Task (c-RST) and children's Melody Discrimination Tasks (c-MDT) were based on two tasks previously used with adults (RST; Chen et al., 2008; MDT, Foster and Zatorre, 2010a). For both tasks, we assessed whether children's patterns of performance would be similar to adults across levels of difficulty, whether performance would be better for children with music training, and whether scores would increase with age. Using the age-normed scores derived from the non-musician sample, we also assessed the contributions of years of music training to performance, and the possible relationships between music and cognitive abilities, including auditory working memory.

Musical ability is defined as the innate potential to perceive, understand, and learn music (Law and Zentner, 2012; Schellenberg and Weiss, 2013). It is assumed that, like other innate capacities, musical abilities are normally distributed in the population (Schellenberg and Weiss, 2013), and that even without musical training these abilities develop with age (Stalinski and Schellenberg, 2012). In the first year, infants can discriminate between simple rhythm patterns and meters (Hannon and Johnson, 2005). Producing synchronized movement takes longer to master. Children as young as four can tap to a beat, and this ability improves between 4 and 11 years old (Drake et al., 2000). Existing evidence shows that by age 7 children can reproduce very short rhythms (Drake, 1993; Drake et al., 2000; Repp and Su, 2013). Children become more sensitive to the metrical structures of their culture with exposure to music (Corrigall and Schellenberg, 2015), and by adulthood are better at detecting changes in rhythms with a metrical structure specific to their culture (Hannon and Trehub, 2005). Basic melody discrimination is in place very early in life. Even before birth, near-term fetuses can detect a change in pitch of roughly an octave (Lecanuet et al., 2000). By 2 months old infants can discriminate between semitones, and they can process transposed songs, a more cognitively demanding task, by early childhood (Plantinga and Trainor, 2005, 2009). The brain's response to auditory stimuli has a relatively long developmental timeframe, continuing to mature until 18–20 years old (Ponton et al., 2002). As children move through the school years they are more sensitive to aspects of music specific to their culture (Corrigall and Schellenberg, 2015). Implicit knowledge of key membership is acquired first, followed by implicit knowledge of harmony (Lynch et al., 1990; Trainor and Trehub, 1994; Schellenberg et al., 2005). Explicit knowledge of key membership and harmony begins around 6 years old and continues to develop until 11 years old (Costa-Giomi, 1999).

School-aged children with musical training—even as little as 1–3 years—have been found to score higher on musical tasks than those with no training. Longitudinal and quasi-experimental studies provide the most compelling evidence for the effects of musical training on musical abilities. Six-year-olds who received 15 months of keyboard lessons improved on a combined melodic and rhythmic discrimination score compared to controls (Hyde et al., 2009). In a sample of children aged 7–8, rhythm and tonal discrimination improved significantly more after 18 months of musical training than after science training (Roden et al., 2014b). In another study, children were followed from ages 7–13; those with music training showed better detection of deviant musical stimuli, as measured with the mismatch negativity ERP response (Putkinen et al., 2013). Most recently, children aged 6–8 were given group music lessons, group soccer training, or no training for 2 years (Habibi et al., 2016). The musically trained children were the most accurate at discriminating changes in pitch.

The earliest tests for measuring children's musical ability included both perceptual tasks such as discriminating among pitches or timbres, and motor tasks such as controlling tempo while singing (Seashore, 1915). Subsequent batteries have focused more on perceptual tasks, perhaps due to the difficulty of administering and evaluating children's musical performance objectively. The most recent and well-known batteries of music perception with age-equivalent scores for school-aged children are the *Primary* and *Intermediate Measures of Music Audiation* (PMMA and IMMA; Gordon, 1979, 1986). The PMMA and IMMA are commonly used in research, given that there are norms for children in different age groups. However, these norms have not been updated for three to four decades. Thus, cohort effects related to changes in music-listening and in cognitive variables known to be related to musical abilities may make these norms less valid for current use (Nettelbeck and Wilson, 2004). More recent test batteries include the Montreal Battery of Evaluation of Musical Abilities (MBEMA; Peretz et al., 2013), which was administered to a large sample of Canadian and Chinese children aged 6–8. Like the PMMA and IMMA, the MBEMA consists of perceptual discrimination tasks (contour, scale, interval, and rhythm), with an added memory task. Although scores are reported for children with up to 2 years of musical training, the test was designed to identify amusia (an auditory-processing deficit), and as such may not be sensitive enough to detect differences in ability between children with and without training, or changes with age. Most recently, researchers developed a battery of tests of music perception, standardized on over 1,000 Brazilian schoolchildren aged 7–13 (Barros et al., 2017). Test scores showed no correlations with age, indicating that the task may not be useful in a developmental context. In addition, no musically-trained children were included in the sample.

In sum, children's musical abilities appear to change with age, and are influenced by musical training. It also appears that, overall, s rhythm synchronization and melody discrimination abilities emerge at different ages, with melodic abilities developing earlier. Further, more modern tests of musical abilities in children may be limited in their utility for examining the effects of development and training. Given the increased interest in assessing musical skills in childhood, an important goal of this study is to provide the community with reliable tests with up-to-date scores accounting for the influence of age.

Cognitive abilities such as working memory and non-verbal reasoning change with age, and are associated with both musical training and with musical aptitude (Schellenberg and Weiss, 2013; Swaminathan et al., 2016). Even after very little training, children score higher on age-equivalent measures of immediate and short-term working memory (Bergman Nutley et al., 2014; Roden et al., 2014a). In a well-known longitudinal study, children's scores on tests of global cognitive function increased after 36 weeks of music lessons, when compared to art lessons or no lessons (Schellenberg, 2004). In addition, there is evidence of associations between musical and language abilities (Patel, 2012; Gordon et al., 2015a). For instance, melody perception and language comprehension are strongly correlated by age 5 (Sallat and Jentschke, 2015), and young children's ability to detect large deviations of pitch in speech were found to improve after only 8 weeks of music lessons (Moreno and Besson, 2006). By age 6, children's rhythmic perceptual abilities are predictive of their ability to produce complex grammatical structures (Gordon et al., 2016). In children with lower SES, small amounts of music lessons may have a protective effect on literacy skills, compared to control subjects (Slater et al., 2014). Given the complex overlap between musical, cognitive, and language skills, and their relation to music training, in the current study we administered tests of auditory working memory and global cognitive function.

**Figure 1:**
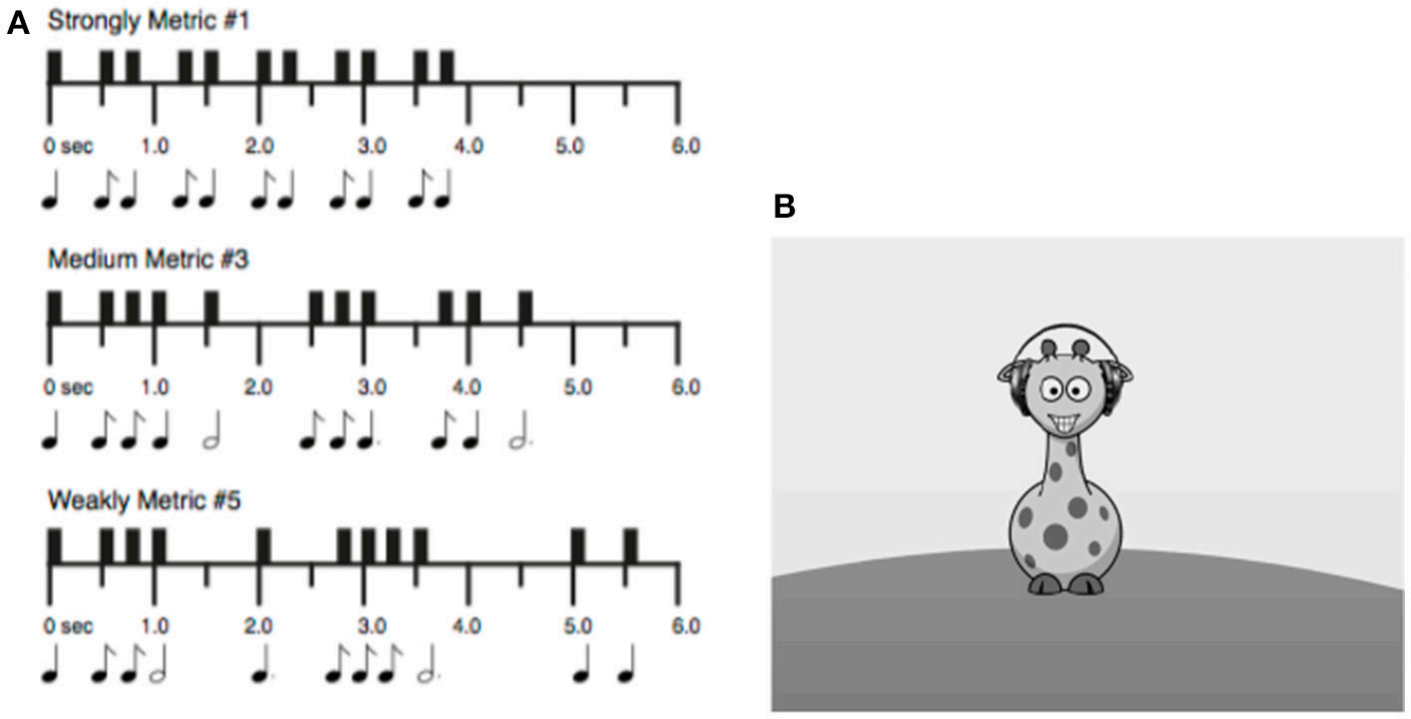
**(A)** Examples of stimuli used in the c-RST. Strongly metric, medium metric, and weakly metric refer to the regularity of the underlying pulse (Strongly metric = easiest). Items were matched for number of notes (11). Figure adapted from Tryfon et al. (2017). **(B)** Graphical display for the c-RST. Image is presented in full color within the actual task.

**Figure 2:**
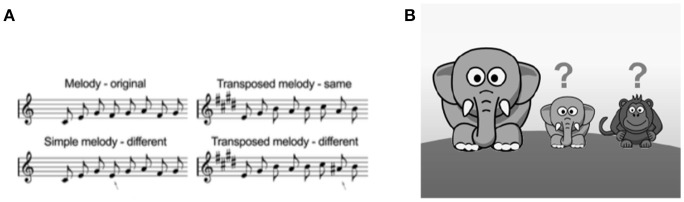
**(A)** Examples of stimuli from the c-MDT Simple Melody condition (L) and transposed melody condition (R). Children listen to two melodies and decide whether the second was the same or different. Arrows represent the “different” note. Melodies range from 5–11 notes; examples are depicted with 8 notes. Figure adapted from Karpati et al. (2016). **(B)** Graphical display of response probe for the c-MDT. Image is presented in full color within the actual task. Small animals represent “same” and “different” response choices.

The tests of musical ability developed for the current study are based on adult tasks. Both tasks were abbreviated and simplified to be more engaging and have a shorter administration time. The children's Rhythm Synchronization Task (c-RST; Figure 1) and children's Melody Discrimination Task (c-MDT: Figure 2) were adapted following guidelines advanced by Corrigall and Schellenberg (2015), including adding a storyline, reducing test duration, and providing feedback.

The Rhythm Synchronization Task (RST) is a computer-based task that assesses the ability to tap in synchrony to a series of rhythms that vary in metrical complexity. It is based on an adult task initially developed for brain imaging and then modified for behavioral studies (Chen et al., 2008). Adult professional musicians scored higher than non-musicians on the RST (Bailey and Penhune, 2010, 2012; Karpati et al., 2016). Moreover, irrespective of training, scores decreased as metric regularity (indicated by the presence of a steady pulse) decreased (Chen et al., 2008; Bailey and Penhune, 2010; Matthews et al., 2016). The RST was recently adapted for children, with the purpose of comparing typically developing children and those with autism spectrum disorder (Tryfon et al., 2017). The Melody Discrimination Task (MDT) is a computer-based task that assesses the ability to discriminate between two melodies that differ by one note either in the same key or transposed. Adult musicians outperformed non-musicians on this task (Foster and Zatorre, 2010a; Karpati et al., 2016) and scores are related to length of musical training (Foster and Zatorre, 2010b). For the current study this task was shortened, and a storyline added, for use with children. Items were selected for optimal reliability and difficulty.

The goal of the present study is to assess the influence of age and musical training on children's musical abilities using the RST and MDT, two tasks widely used with adults. Considering the different paradigms of these two tasks (i.e., RST, a production task, and MDT, a perceptual task), and the likely differences in developmental trajectories of the rhythmic and melodic abilities measured, we assess rhythm and melody separately. We provide standardized scores for each age group, and use these scores to investigate the effects of musical training on task performance. Finally, we assess the relation between musical, baseline and cognitive abilities in musically trained children.

## Materials and methods

### Participants

We tested 213 children aged 7–13 years in music and science camps in Montréal, Ottawa, and Waterloo, Canada. Children were categorized as musicians (*n* = 130) or non-musicians (*n* = 83) based on a parent questionnaire adapted in our lab (Survey of Musical Interests; Desrochers et al., 2006). The term musician was operationalized as a child who had at least 2.5 years of consecutive music lessons (*M* = 5.06 years, *SD* = 1.58, range 2.74–10.00). Music lessons were operationalized as extra-curricular, weekly, one-on-one sessions of at least 30 min in duration and taught by an expert. Child musicians also practiced for at least half an hour a week (*M* = 3.16 h, *SD* = 2.49, range = 0.50–14.00). Music practice could be structured (using a book or specific exercises) or unstructured (free playing), as long as it occurred outside of lessons and on the same instrument. The term non-musician was operationalized as a child with no more than 2.5 years of consecutive lessons (*M* = 0.43, *SD* = 0.74, range 0.00–2.30). We assessed children's SES by estimating maternal years of education. As in the original questionnaire, mothers reported their highest level of education on an ordinal scale. We converted this to an approximate interval scale with the following estimates: high school = 12 years; college diploma = 14 years; baccalaureate degree = 16 years; master's degree = 18 years; doctorate or medical professional degree = 22 years.

Demographic and practice-related characteristics for all children by musicianship and age group are in given in Table 1. Parents provided written consent and children provided verbal assent before participating. Children were given a gift card and a small toy as thanks for their participation. The study was approved by Concordia University's Human Research Ethics Board.

**Table 1:** Demographic and practice characteristics of the sample (*N* = 213), by musicianship and age group.

**Musicians (*n* = 130)**	**7**	**8**	**9**	**10**	**11**	**13**
	***n* = 11 (6F)**	***n* = 18 (13F)**	***n* = 23 (15F)**	***n* = 24 (12F)**	***n* = 30 (16F)**	***n* = 24 (18F)**
Age (years)	7.59 (0.41)	8.46 (0.26)	9.50 (0.33)	10.46 (0.28)	11.69 (0.40)	13.00 (0.49)
Maternal education (years)	17.27 (2.87)	18.53 (2.77)	18.29 (2.31)	18.17 (2.33)	17.00 (2.61)	16.94 (2.24)
Age of start (years)	4.13 (0.70)	4.44 (0.64)	4.71 (1.23)	5.29 (1.31)	5.71 (1.43)	7.69 (1.43)
Music lessons (years)	3.42 (0.73)	4.01 (0.66)	4.67 (1.29)	5.04 (1.31)	5.93 (1.55)	5.27 (1.45)
Weekly practice (hours)	2.52 (2.15)	3.17 (1.79)	2.83 (1.59)	3.24 (1.73)	3.56 (3.06)	2.32 (2.26)
**Non-musicians (*****n*** = **83)**	**7**	**8**	**9**	**10**	**11**	**13**
	***n*** = **15 (5F)**	***n*** = **14 (6F)**	***n*** = **16 (7F)**	***n*** = **13 (7F)**	***n*** = **13 (7F)**	***n*** = **12 (5F)**
Age (years)	7.09 (0.45)	8.51 (0.36)	9.46 (0.29)	10.46 (0.22)	11.66 (0.45)	13.65 (0.57)
Maternal education (years)	17.60 (2.16)	17.66 (2.70)	18.40 (2.33)	16.22 (1.28)	18.15 (2.51)	15.56 (0.75)

### Rhythm synchronization task

The child version of the RST (c-RST; Figure 1) differs from the adult task in several ways (Tryfon et al., 2017). First, to make it more engaging, a storyline and corresponding graphics were generated. Next, task difficulty was reduced by removing the most difficult (“non-metric”) rhythm level, and replacing it with an easy (“strongly metric”) level. Thus, the c-RST has three levels of rhythmic complexity that vary in difficulty from easiest to hardest: Strongly Metric, Medium Metric, and Weakly Metric. There are two rhythms per difficulty level, for a total of six rhythms which are presented in counterbalanced order. Rhythms were matched for number of notes; each rhythm consists of 11 woodblock notes spanning an interval of 4–5.75 s, including rests. As with the adult task, a single trial of the c-RST consists of two phases: (1) “Listen” and (2) “Tap in Synchrony.” In the graphical display, a giraffe with headphones is displayed on the computer screen. During the Listen phase, the giraffe's headphones are highlighted, indicating that the child should listen to the rhythm without tapping. During the Tap in Synchrony phase, the giraffe's hoof is highlighted, indicating that the child should tap along in synchrony with each note of the rhythm using the index finger of the right hand on a computer mouse. Each of the six rhythms is presented for three trials in a row, for a total of 18 trials. Before starting the test, children complete five practice trials at the Strongly Metric level, with feedback from the experimenter. The rhythms used for the practice trials are not those used in the main task. Performance on the RST is measured in two outcomes: (1) percent correct, or the child's ability to tap within the “scoring window” (as explained below); and (2) percent inter-tap interval (ITI) synchrony, or the child's ability to reproduce the temporal structure of a rhythm. The percent correct is calculated as the proportion of taps that fall within the scoring window (i.e., half the interval before and after the stimulus). The ITI synchrony is calculated as the ratio of the child's response intervals (r) to the stimulus time intervals (t), with the following formula: Score = 1–abs(r–t)/t. For both percent correct and ITI synchrony, proportions are multiplied by 100 to generate a percentage.

### Tapping and continuation task

The Tapping and Continuation Task has been used in both adults and children to measure basic synchronization and timing abilities that do not differ between those with and without musical training (Aschersleben, 2002; Balasubramaniam et al., 2004; Whitall et al., 2008; Corriveau and Goswami, 2009; Tierney A. and Kraus, 2013; Matthews et al., 2016; Dalla Bella et al., 2017; Tryfon et al., 2017). The ability to synchronize to a beat has also been found to relate to general cognitive domains such as language and attention (Tierney A. T. and Kraus, 2013). Thus, the TCT may serve as an auditory-motor and cognitive control task for the RST. For this task, children tap along with an isochronous rhythm of woodblock notes for 15 s (paced tapping), and are instructed to continue tapping at the same tempo for 15 s once the rhythm stops (non-paced tapping). The tapping task runs for six trials at the same tempo [inter-stimulus interval (ISI) of 500 ms]. Performance is measured in terms of tapping variability; paced and non-paced trials are scored separately. The ITIs and their respective standard deviations are averaged across all six trials for paced and non-paced tapping. The average *SD* is then divided by the average ITI to generate a coefficient of variation (i.e., the child's tapping variability relative to his or her own performance).

### Melody discrimination task

For each trial of the MDT, participants listen to two melodies of equal duration separated by a 1.2-s silence, and then indicate whether the second melody is the same or different than the first. There are two conditions: Simple and Transposed. In the Simple condition, both melodies are in the same key. In the “different” trials, the pitch of a single note in the second melody is shifted up or down by up to five semitones, while preserving the contour of the first melody. The participant thus must compare individual pitches to detect the deviant note. In the Transposed condition, all the notes in the second melody are transposed upward by four semitones (a major third). In the “different” trials a single note is shifted up or down by one semitone, while preserving the contour of the first melody. Thus, the participant must use relative pitch to perceive the deviant note within a transposed model. All melodies in the MDT were composed of low-pass-filtered isochronous harmonic tones (320 ms each, corresponding to a tempo of 93.75 bpm) from the Western major scale, using tones taken from the two octaves between C4-E6. All major scales are represented except B, F-sharp, and C-sharp; minor scales include E, A, and E-flat.

The child version of the MDT (c-MDT; Figure 2) differs from the adult version in several ways. The adult version comprises 180 melodies (90 simple and 90 transposed), which range from 5 to 13 notes per melody. This was considered too long for testing with children so 60 items were selected (30 simple and 30 transposed) based on a reduced range of notes for lower difficulty (5–11 notes per melody). After this set of 60 items was administered to all children, we calculated item-level statistics *post-hoc* in order to retain a “best set” of data with the following criteria: (1) KR-20, or Cronbach's alpha for dichotomous items, of at least 0.50; (2) point-biserial correlation, or the degree to which items correlate with the total score for each condition, of at least 0.10; (3) item difficulty above chance; and (4) administration time under 20 min, including instructions and practice. The resulting best set is composed of 40 melodies, 20 per condition, with 5–11 notes per melody. The results reported in the current paper are for this best set. Raw score means and standard deviations for the 60-item set are provided for comparison in the Appendix in Supplementary Material.

The Simple and Transposed conditions each have 20 trials, with an equal number of “same” and “different” trials per condition. Each condition is presented as two blocks of 10 trials with a break in between. The 20 trials are presented in random order within conditions, but the order of conditions is always the same (Simple, Transposed) to preserve the storyline. In the corresponding graphical display, children see a teacher elephant who “sings” a melody which is then repeated by either the “echoing elephant who sings it perfectly” or the “forgetful monkey who always makes a little mistake.”

In the graphical display for the Transposed condition, children are again shown the teacher elephant who sings the melody, which is repeated by the “baby elephant” or the “baby monkey” who “sing in a much higher voice” (i.e., in a transposed key); they are instructed to ignore this difference and instead listen for the “little mistake.”

### Syllable sequence discrimination task

The Syllable Sequence Discrimination Task (SSDT) was designed as a baseline task for the MDT that would place similar demands on auditory working memory ability. In the c-SSDT the child hears two sequences of 5–8 non-word syllables, spoken in a monotone with F0 held constant, and judges whether they are the same or different. Syllables were generated using permutations of 7 consonants [f, k, n, p, r, s, y] and 4 vowel sounds [a, i, o, u], which were then selected for minimal semantic association (Foster and Zatorre, 2010a). The c-SSDT contains the following 13 phonemes: fah, foh, foo, kah, koh, nah, poh, rah, ree, roh, roo, sah, yah. Sequence lengths (5–8 syllables) were selected to match the adult version of the task. In the graphical display adapted for this task, the elephant and monkey are shown wearing robot helmets and are said to be “copying robot sounds,” with the same response cue as in the c-MDT (“echoing elephant” or “forgetful monkey”).

For both the c-MDT and c-SSDT, children are familiarized through four practice trials with the experimenter watching. Feedback is provided on the first two of these practice trials to ensure the child understands the task. After all trials, the word “correct” or “incorrect” is displayed for 1 s. Experimenters are seated so as not see children's responses or feedback during experimental trials. Discrimination is scored as the percentage of correct responses. The child's responses are scored as 0 (incorrect) or 1 (correct), generating a proportion which is then multiplied by 100.

### Cognitive tasks

To assess cognitive abilities that might be related to performance on the music tasks we administered the Digit Span (DS), Letter-Number Sequencing (LNS), and Matrix Reasoning (MR) subtests from the Wechsler Intelligence Scale for Children, fourth edition (WISC-IV; Wechsler, 2003). Digit Span is a measure of immediate auditory memory, in which the child repeats strings of digits forward or backward. Letter-Number Sequencing (LNS) is a measure of auditory working memory and manipulation, in which the child hears a string of letters and numbers and must repeat them back in numerical and alphabetical order, respectively. Matrix Reasoning (MR) is a measure of non-verbal reasoning, and is considered to be a reliable estimate of general intellectual ability (Brody, 1992; Raven et al., 1998). For this task, the child must identify the missing portion of an incomplete visual matrix from one of five response options.

All subtests were administered according to standardized procedures. Raw scores were converted to scaled scores based on age-based norms for all three subtests. The population-based mean for subtest scaled scores on the WISC-IV is 10, with a standard deviation of 3 (Wechsler, 2003).

### General procedure

Testing took place over a 1-h session. Participants were given short breaks between tasks to enhance motivation. Computer-based tasks were administered on a laptop computer running Presentation software (Neurobehavioral Systems, http://www.neurobs.com/). Auditory tasks were presented binaurally via Sony MDRZX100B headphones adjusted to a comfortable sound level. Musical tasks were administered before cognitive tasks, with musical task order (either c-RST or c-MDT first) counterbalanced across participants. Cognitive tasks were administered in the order in which they appear in the original WISC-IV battery.

All programs for administration and scoring, as well as a user manual with norms, will be made available upon request to the first author.

## Results

### Sample characteristics: child musicians and non-musicians

Data for group differences in the sample are presented in Table 2.

**Table 2:** Group differences between musicians and non-musicians on demographic, baseline, and cognitive tasks.

**Measure**	**Musicians (SD)**	**Non-musicians (SD)**	***t (df)***	***p***	***g***
Maternal education (years)	17.54 (2.44)	17.34 (2.28)	0.59 (211)	0.558	0.08
TCT (paced variability)	0.12 (0.02)	0.12 (0.02)	−0.72 (211)	0.472	0.11
TCT (non-paced variability)	0.10 (0.05)	0.13 (0.05)	3.49 (211)	0.001	0.49
c-SSDT (percent correct)	82.17 (12.36)	76.04 (12.73)	−4.83 (211)	<0.001	0.68
DS (scaled score)	11.45 (3.08)	10.68 (2.94)	1.79 (207)	0.076	0.26
LNS (scaled score)	11.68 (1.92)	11.47 (2.18)	0.75 (207)	0.454	0.10
MR (scaled score)	12.44 (2.62)	11.53 (2.84)	2.35 (207)	0.020	0.33

*TCT, tapping and continuation task (baseline); c-SSDT, children's syllable sequence discrimination task (baseline); DS, digit span; LNS, letter-number sequencing; MR, matrix reasoning*.

We first conducted a chi-square analysis to determine whether the number of boys and girls differed between musicians and non-musicians. There were significantly more female musicians than males, and significantly more male non-musicians than females [$\chi_{\left(1\right)}^{2}$ = 5.89, *p* = 0.015]. Subsequently we carried out ANOVAs with musicianship and gender as between-subjects factors. For Simple melodies there was a small but statistically significant musicianship-by-gender interaction [*F*_(1, 209)_ = 5.53, *p* = 0.02, partial η^2^ = 0.03)], such that the difference between male musicians and non-musicians (20%) was greater than the difference between female musicians and non-musicians (12%). However, there were no such interactions for any other outcome variables of interest for either the c-RST or c-MDT. Thus, gender was not added as a covariate for group difference analyses.

We conducted independent-sample *t*-tests, and calculated Hedge's *g* effect sizes, to examine the degree to which musicians and non-musicians differed in SES (estimated years of maternal education), cognitive variables including auditory working memory (Digit Span, LNS) and general intellectual ability (Matrix Reasoning), or performance on baseline tasks (Paced and Non-paced Tapping Variability, Syllable Sequence Discrimination). Cognitive data were lost for four children but as they represent less than 5% of the sample these scores were not replaced (Kline, 2011). Twelve musician's mothers and 10 non-musician's mothers did not answer the question about maternal education.

There were no statistically significant differences between musician's and non-musician's SES [*t*_(189)_ = 0.43, *p* = 0.67, *g* = 0.06]. Likewise, there were no differences in auditory working memory [DS *t*_(207)_ = 1.79, *p* = 0.08, *g* = 0.25; LNS *t*_(207)_ = 0.75, *p* = 0.45, *g* = 0.10]. Although statistically different, both groups scored in the Average range for general intellectual ability [Matrix Reasoning *t*_(207)_ = 2.28, *p* = 0.023, *g* = 0.32.]. Overall, children scored at or above the population mean on all cognitive tasks, but were likely to have higher SES than in the stratified normative sample of the WISC-IV. For performance on baseline tasks, there were no differences in the TCT (paced tapping) [*t*_(211)_ = −0.72, *p* = 0.472, *g* = 0.10]. However, musicians had significantly better performance in the TCT (non-paced tapping) [*t*_(211)_ = −3.86, *p* < 0.001, *g* = 0.54] and Syllable Sequence Discrimination tasks [*t*_(211)_ = 3.49, *p* = 0.001, *g* = 0.48]. Therefore, these were included as covariates for the regression analyses.

### Reliability

To examine internal-consistency reliability, we used Cronbach's alpha for the c-RST, which estimates the mean of all possible split-half reliabilities, and KR-20 for the c-MDT, equivalent to Cronbach's alpha for dichotomous variables. Reliability estimates were derived for musicians and non-musicians separately. Scores on the c-RST were found to be adequately reliable for musicians (α = 0.64) but slightly less so for non-musicians (α = 0.60). Score reliability is higher on the c-MDT and, similar to the c-RST, is higher for musicians (KR-20 = 0.86) than for non-musicians (KR-20 = 0.75).

### Effects of musicianship, task, and age

To examine the degree to which performance on the c-RST and c-MDT varied between musicians and non-musicians, across levels of each task (e.g., rhythmic complexity and melody type), and between children of different age groups, we carried out mixed-design ANOVAs. We included musicianship (musician or non-musician) and age group (7, 8, 9, 10, 11, 13) as between-subjects factors, and task level as a repeated measure (c-RST: Strongly Metric, Medium Metric, Weakly Metric; c-MDT: Simple Melodies, Transposed Melodies). Outcome variables for the c-RST were percent correct and ITI synchrony; the outcome for the c-MDT was percent correct. Partial eta-squared effect sizes were calculated, and *post-hoc* analyses were carried out with Bonferroni corrections for multiple comparisons.

For the c-RST (percent correct and ITI synchrony), the assumption of sphericity was violated such that the variances of the differences between levels of rhythmic complexity were not homogeneous (Mauchly's *W* = 0.94, *p* = 0.002 for both). Thus, degrees of freedom for all effects were corrected using Greenhouse-Geisser estimates ($\hat{\varepsilon \;}=$ 0.94 for percent correct and 0.95 for ITI synchrony).

For the c-RST—percent correct, there was a marginally significant effect of musicianship [*F*_(1, 201)_ = 3.65, *p* = 0.058, partial η^2^ = 0.02], and significant main effects of rhythmic complexity [*F*_(1.89, 379.64)_ = 205.24, *p* < 0.001, partial η^2^ = 0.51] and age group [*F*_(5, 201)_ = 5.24, *p* < 0.001, partial η^2^ = 0.12]. Overall, children's scored taps decreased in a stepwise fashion from Strongly Metric to Medium Metric (*p* < 0.001), and from Medium Metric to Weakly Metric rhythms (*p* = 0.004). *Post-hoc* comparisons revealed that the oldest children outperformed the youngest but there were no stepwise changes between age groups. There was a significant interaction between rhythmic complexity and age [*F*_(9.44, 379.64)_ = 4.48, *p* < 0.001, partial η^2^ = 0.10]. Decomposition of this interaction revealed that children's scored taps increased significantly more with age for Strongly Metric rhythms than for the more difficult rhythms.

For the c-RST – ITI synchrony, there were significant main effects of musicianship [*F*_(1, 201)_ = 9.39, *p* = 0.002, partial η^2^ = 0.05], rhythmic complexity [*F*_(1.89, 379.71)_ = 250.95, *p* < 0.001, partial η^2^ = 0.56], and age group [*F*_(5, 201)_ = 12.13, *p* < 0.001, partial η^2^ = 0.23]. Overall, musicians outperformed non-musicians and synchronization ability decreased in a stepwise fashion from Strongly Metric to Medium Metric (*p* < 0.001), and from Medium Metric to Weakly Metric rhythms (*p* = 0.005). *Post-hoc* comparisons revealed that the oldest children tapped more in synchrony than the youngest, but there were no stepwise changes between age groups. There was also a significant age-group-by-complexity interaction [*F*_(9.45, 379.71)_ = 2.27, *p* = 0.016, partial η^2^ = 0.05], such that scores differed the most with age for Strongly Metric rhythms.

For the c-MDT, significant main effects were found for musicianship [*F*_(1, 198)_ = 76.01, *p* < 0.001, η^2^ = 0.28], melody type [*F*_(1, 198)_ = 141.31, *p* < 0.001, η^2^ = 0.42], and age group [*F*_(5, 198)_ = 5.90, *p* = 0.001, η^2^ = 0.13]. No significant interaction effects were found. Overall, musicians scored higher than non-musicians and children's scores were higher for Simple melodies than Transposed melodies. *Post-hoc* comparisons revealed that, overall, the oldest children performed best, but there were no significant stepwise increases between age groups.

### Age-equivalent scores

Given the main effects of age group for both the c-RST and c-MDT, we created age-equivalent (z-) scores for children on each task and their respective baseline tasks (c-TST and c-SSDT), using the formula z = (raw score—age group mean)/age group standard deviation. Means and standard deviations were derived from non-musicians (*n* = 83), who serve as the reference group with very little or no musical experience. Raw score means and standard deviations for musicians and non-musicians are presented in Table 3 (with the 40-item version of the c-MDT reported), and z-score conversions are provided in Table 4. Based on these, researchers using the c-RST or c-MDT with new groups of children can compare performance to either the trained or untrained sample.

**Table 3:** Raw score means and standard deviations for music and baseline tasks, by musicianship and age group.

**MUSICIANS**	**7**	**8**	**9**	**10**	**11**	**13**
**c-RST: Percent Correct**	***n*** = **11 (6F)**	***n*** = **18 (13F)**	***n*** = **23 (15F)**	***n*** = **24 (12F)**	***n*** = **30 (16F)**	***n*** = **24 (18F)**
Strongly metric	83.18 (6.10)	86.17 (7.13)	89.26 (6.37)	89.50 (7.22)	92.80 (4.66)	92.96 (5.86)
Medium metric	75.18 (5.67)	76.50 (5.18)	80.30 (6.76)	79.83 (7.53)	82.80 (6.73)	80.67 (6.66)
Weakly metric	74.64 (5.45)	77.17 (6.19)	79.52 (3.80)	78.00 (8.02)	81.73 (4.68)	76.83 (7.23)
**c-RST: ITI Synchrony**						
Strongly metric	70.82 (11.33)	73.94 (9.94)	77.48 (9.64)	80.54 (7.33)	82.77 (8.08)	83.96 (4.86)
Medium metric	60.18 (7.85)	59.78 (9.68)	57.74 (6.39)	63.33 (9.41)	67.67 (6.73)	67.33 (6.03)
Weakly metric	57.36 (8.82)	53.61 (11.48)	62.74 (9.09)	60.75 (9.56)	64.83 (7.21)	63.79 (6.05)
**TCT: Paced Variability**	0.13 (0.02)	0.11 (0.02)	0.11 (0.02)	0.13 (0.03)	0.12 (0.02)	0.11 (0.01)
**TCT: Non-paced Variability**	0.14 (0.05)	0.11 (0.06)	0.11 (0.06)	0.10 (0.05)	0.08 (0.02)	0.09 (0.03)
**c-MDT: Percent Correct**						
Simple melodies	72.27 (18.35)	78.61 (10.40)	81.30 (10.79)	81.46 (11.84)	86.00 (8.55)	80.00 (12.34)
Transposed melodies	58.18 (11.24)	58.89 (13.67)	68.70 (13.50)	65.42 (17.63)	71.67 (10.37)	69.13 (15.35)
**c-SSDT: Percent Correct**	74.09 (13.93)	81.67 (14.35)	80.22 (13.44)	80.00 (12.85)	86.83 (8.46)	85.43 (11.47)
**NON-MUSICIANS**	**7**	**8**	**9**	**10**	**11**	**13**
**c-RST: Percent Correct**	***n*** = **15 (5F)**	***n*** = **14 (6F)**	***n*** = **16 (7F)**	***n*** = **13 (7F)**	***n*** = **13 (7F)**	***n*** = **12 (5F)**
Strongly metric	80.07 (10.14)	85.00 (9.51)	86.00 (8.22)	84.62 (10.12)	87.54 (6.98)	94.75 (3.49)
Medium metric	78.20 (6.33)	77.00 (5.04)	78.81 (9.20)	77.69 (5.59)	81.15 (3.51)	79.50 (6.54)
Weakly metric	77.53 (8.41)	77.07 (8.15)	76.69 (8.78)	75.85 (6.67)	77.62 (7.62)	75.92 (7.45)
**c-RST: ITI Synchrony**						
Strongly metric	68.73 (10.26)	68.79 (26.31)	76.31 (10.00)	75.15 (8.87)	81.69 (5.50)	86.83 (3.54)
Medium metric	56.87 (4.91)	56.07 (7.47)	63.87 (7.73)	58.23 (8.70)	60.38 (8.39)	66.08 (9.61)
Weakly metric	54.13 (16.73)	52.36 (12.00)	58.69 (12.47)	58.85 (8.66)	60.46 (10.46)	54.17 (15.63)
**TCT: Paced Variability**	0.12 (0.02)	0.12 (0.02)	0.13 (0.02)	0.12 (0.02)	0.11 (0.02)	0.11 (0.01)
**TCT: Non-paced Variability**	0.16 (0.04)	0.13 (0.05)	0.15 (0.09)	0.12 (0.05)	0.11 (0.02)	0.09 (0.04)
**c-MDT: Percent Correct**						
Simple melodies	57.00 (12.07)	67.50 (12.05)	61.56 (10.12)	69.62 (16.00)	65.00 (11.37)	74.17 (18.07)
Transposed melodies	49.67 (7.43)	50.71 (11.91)	50.63 (9.29)	52.31 (11.66)	53.46 (9.22)	60.00 (11.28)
**c-SSDT: Percent Correct**	72.33 (14.13)	67.14 (9.55)	78.75 (10.88)	83.46 (11.44)	82.69 (11.48)	76.25 (14.00)

*c-RST, children's rhythm synchronization task; TCT, tapping and continuation task (baseline); c-MDT, children's melody discrimination task; c-SSDT, children's syllable sequence discrimination task (baseline)*.

**Table 4:** Raw score to age-equivalent (Z-) score conversion table for music and baseline task outcome variables, by musicianship.

	**c-RST: % corr (Strong)**	**c-RST: % corr (Medium)**	**c-RST: % corr (Weak)**	**c-RST: ITI synch (Strong)**	**c-RST: ITI synch (Medium)**	**c-RST: ITI synch (Weak)**	**TCT: Variability (Paced)**	**TCT: Variability(Non-paced)**	**c-MDT: % corr (Simple)**	**c-MDT: % corr (Transposed)**	**c-SSDT: % corr**	
**MUSICIANS**												
**z**												**z**
+3.0	100.00	96.69	92.45	92.00	83.07	81.00	0.08	0.04	100.00	95.00	100.00	+3.0
+2.5	98.76	94.76	89.00	92.00	80.38	78.52	0.08	0.04	100.00	95.00	100.00	+2.5
+2.0	97.14	91.00	88.00	89.14	77.00	74.14	0.09	0.05	100.00	90.00	100.00	+2.0
+1.5	97.00	86.00	85.00	87.00	71.04	70.04	0.09	0.05	95.00	85.00	95.00	+1.5
+1.0	95.00	83.00	82.00	85.00	68.00	67.00	0.10	0.07	90.00	75.00	90.00	+1.0
+0.5	91.00	80.00	79.00	82.00	64.00	63.00	0.10	0.08	80.00	67.00	85.00	+0.5
0	88.00	77.00	76.00	78.00	60.00	58.00	0.11	0.09	75.00	60.00	75.00	0
−0.5	83.00	74.00	73.00	70.00	54.96	51.96	0.12	0.10	69.80	50.00	70.00	−0.5
−1.0	76.00	68.00	65.00	60.00	49.00	45.00	0.14	0.13	60.00	44.30	60.00	−1.0
−1.5	70.62	63.48	62.10	54.24	44.00	39.10	0.16	0.18	46.20	38.10	45.00	−1.5
−2.0	70.00	58.93	56.24	53.00	41.24	35.31	0.17	0.28	36.55	35.00	45.00	−2.0
−2.5	70.00	58.00	55.00	53.00	40.00	35.00	0.22	0.30	35.00	35.00	45.00	−2.5
−3.0	–	–	–	–	–	–		–	–	–	–	−3.0
**NON-MUSICIANS**												
**z**												**z**
+3.0	–	–	–	–	–	–	–	–	–	–	–	+3.0
+2.5	–	–	–	–	–	–	0.08	0.04	–	–	–	+2.5
+2.0	–	–	–	–	–	–	0.08	0.05	–	–	-	+2.0
+1.5	98.64	90.60	92.00	92.32	81.64	76.64	0.09	0.07	96.60	75.00	100.00	+1.5
+1.0	98.00	88.00	88.96	89.00	74.96	73.96	0.10	0.09	85.00	70.00	95.00	+1.0
+0.5	95.00	83.00	85.00	87.00	69.00	69.56	0.11	0.11	80.00	65.00	90.00	+0.5
0	92.00	82.00	79.12	82.24	64.00	63.00	0.11	0.13	75.00	60.00	85.00	0
−0.5	88.00	79.00	77.00	79.00	60.00	59.00	0.12	0.15	65.00	50.00	75.00	−0.5
−1.0	82.00	77.00	74.00	73.88	55.00	51.00	0.14	0.16	55.00	45.00	70.00	−1.0
−1.5	77.00	73.00	68.32	64.00	52.00	43.44	0.16	0.20	50.00	45.00	62.20	−1.5
−2.0	70.00	65.20	64.04	59.04	49.04	35.04	0.17	0.30	40.04	33.08	55.00	−2.0
−2.5	60.72	59.76	57.04	28.56	43.44	19.52	–	–	40.00	30.00	48.40	−2.5
−3.0	58.00	55.00	55.00	–	38.00	10.00	–	–	40.00	30.00	45.00	−3.0

*c-RST, children's rhythm synchronization task; % corr, percent correct; ITI synch, inter-tap interval synchrony; Strong, Strongly metric rhythms; Medium, medium metric rhythms; Weak, weakly metric rhythms; TCT, tapping and continuation task; c-MDT, children's melody discrimination task; Simple, simple melodies; Transposed, transposed melodies; c-SSDT, children's syllable sequence discrimination task*.

To examine the contribution of years of training to performance on the c-RST and c-MDT, we conducted hierarchical multiple regressions for all children with at least 1 year of lessons (*n* = 151; Tables 5–8). Outcome variables were z-scores for the c-RST (percent correct and ITI synchrony) and c-MDT (Simple and Transposed melodies). The predictor variable for all three analyses was duration of lessons in years. Scores for the two baseline variables (Non-paced Tapping Variability and Syllable Sequence Discrimination) were entered at the first step, since these were statistically significantly better in musicians.

**Table 5:** Summary of hierarchical regression for baseline and training variables predicting z-scores on the c-RST (percent correct).

**Variable**	**β**	***t***	***R***	***Adj. R^2^***	**Δ*R^2^***
**Step 1**			0.24	0.05	0.06
TCT (z)	0.07	0.84			
c-SSDT (z)	0.23	2.87^**^			
**Step 2**			0.28	0.04	0.00
TCT (z)	0.07	0.83			
c-SSDT (z)	0.23	2.85^**^			
Lessons (years)	0.01	0.07			

*N = 151 (children with > = 1 year of music lessons). The c-RST. children's rhythm synchronization task; TCT (z), tapping and continuation task (non-paced variability) z-score; c-SSDT (z), children's syllable sequence discrimination task z-score. For Step 1, F_(2, 148)_ = 4.56, p = 0.012. For Step 2, F_(1, 147)_ = 0.01, p = 0.943*.

** *p < 0.01*.

**Table 6:** Summary of hierarchical regression for baseline and training variables predicting z-scores on the c-RST (ITI synchrony).

**Variable**	**β**	***t***	***R***	***Adj. R^2^***	**Δ*R^2^***
**Step 1**			0.12	0.00	0.02
TCT (z)	0.02	0.18			
c-SSDT (z)	0.12	1.47			
**Step 2**			0.27	0.05	0.06
TCT (z)	−0.02	−0.19			
c-SSDT (z)	0.14	1.76			
Lessons (years)	0.24	2.99^**^			

*N = 151 (children with > = 1 year of music lessons). The c-RST, children's rhythm synchronization task; TCT (z), tapping and continuation task (non-paced variability) z-score; c-SSDT (z), children's syllable sequence discrimination task z-score. For Step 1, F_(2, 148)_ = 1.11, p = 0.334. For Step 2, F_(1, 147)_ = 8.93, p = 0.003*.

** *p < 0.01*.

**Table 7:** Summary of hierarchical regression for baseline and training variables predicting z-scores on the c-MDT (simple melodies).

**Variable**	**β**	***t***	***R***	***Adj. R^2^***	**Δ*R^2^***
**Step 1**			0.24	0.04	0.06
TCT (z)	−0.02	−0.19			
c-SSDT (z)	0.24	2.98^**^			
**Step 2**			0.33	0.09	0.05
TCT (z)	−0.04	−0.56			
c-SSDT (z)	0.26	3.29^**^			
Lessons (years)	0.23	2.94^**^			

*N = 151 (children with > = 1 year of music lessons). The c-MDT, children's melody discrimination task; TCT (z), tapping and continuation task (non-paced variability) z-score; c-SSDT (z), children's syllable sequence discrimination task z-score. For Step 1, F_(2, 148)_ = 4.44, p = 0.013. For Step 2, F_(1, 147)_ = 8.61, p = 0.004*.

** *p < 0.01*.

**Table 8:** Summary of hierarchical regression for baseline and training variables predicting z-Scores on the c-MDT (transposed melodies).

**Variable**	**β**	***t***	***R***	***Adj. R^2^***	**Δ *R^2^***
**Step 1**			0.18	0.02	0.03
TCT (z)	0.00	0.02			
c-SSDT (z)	0.18	2.28^*^			
**Step 2**			0.39	0.13	0.12
TCT (z)	−0.04	−0.54			
c-SSDT (z)	0.21	2.79^**^			
Lessons (years)	0.35	4.50^***^			

*N = 151 (children with > = 1 year of music lessons). The c-MDT, children's melody discrimination task; TCT (z), tapping and continuation task (non-paced variability) z-score; c-SSDT (z), children's syllable sequence discrimination task z-score. For Step 1, F_(2, 148)_ = 2.59, p = 0.078. For Step 2, F_(1, 147)_ = 20.28, p < 0.001*.

* p < 0.05;

** p < 0.01;

*** *p < 0.001*.

For the c-RST—percent correct, the regression model with only baseline variables accounted for 4.5% of the variance and was statistically significant (adjusted *R*^2^ = 0.05, *p* = 0.012). Additional years of training accounted for no additional variance (adjusted *R*^2^ = 0.04, *p* = 0.943).

For the c-RST—ITI synchrony, the model with only baseline variables was not statistically significant (adjusted *R*^2^ = 0.001, *p* = 0.334). When years of lessons were added, these accounted for 5.6% of the variance and the model was significant (adjusted *R*^2^ = 0.05; *p* = 0.003). Specifically, a one-year increase in lessons contributed to an increase of 0.24 standard deviations in ITI synchrony z-scores (β = 0.24, *p* = 0.003). This is equivalent to a raw-score increase of 1.5% in children without musical training.

For the c-MDT—Simple melodies, the model with only baseline variables was statistically significant (adjusted *R*^2^ = 0.04, *p* = 0.013), and additional years of training accounted for 5.2% additional variance (adjusted *R*^2^ = 0.09, *p* = 0.004). Specifically, a one-year increase in lessons contributed to an increase of 0.23 standard deviations in Simple melody z-scores (β = 0.23, *p* = 0.004). This is equivalent to a raw-score increase of 2.5% in children without musical training.

For the c-MDT—Transposed melodies, the model with only baseline variables was not statistically significant (adjusted *R*^2^ = 0.02, *p* = 0.078). Additional years of training accounted for 11.7% additional variance (adjusted *R*^2^ = 0.13, *p* < 0.001). Specifically, a one-year increase in lessons contributed to an increase of 0.35 standard deviations in Transposed melody z-scores (β = 0.35, *p* < 0.001). This is equivalent to a raw-score increase of 2.9% in children without musical training.

### Relation between musical and cognitive abilities

To examine how musical and baseline tasks relate to cognitive task performance in musicians, we calculated bivariate correlations between age-corrected scores for the seven musical and baseline tasks (c-RST percent correct and ITI synchrony; TCT paced and non-paced tapping variability; c-MDT Simple and Transposed melodies; c-SSDT) and the three cognitive tasks (Digit Span, LNS, and Matrix Reasoning). Given the ample prior evidence that musical training and cognitive variables are positively correlated, bivariate correlations are reported at the one-tailed level of significance. Bonferroni corrections were applied to account for multiple correlations, with a resulting cutoff value of α = 0.002. Zero-order correlations are presented in Table 9.

**Table 9:** Zero-order correlations for music and baseline tasks with cognitive variables in musicians.

**Measure**	**DS**	**LNS**	**MR**
c-RST: % corr (z)	0.22	0.13	0.04
c-RST: ITI synch (z)	0.40^*^	0.33^*^	0.16
TCT: paced (z)	−0.09	−0.09	−0.04
TCT: non-paced (z)	−0.07	−0.12	0.02
c-MDT: Simple (z)	0.16	0.11	0.12
c-MDT: Transposed (z)	0.14	0.15	0.20
c-SSDT (z)	0.33^*^	0.22	0.24

N = 130. Correlations are reported at the one-tailed level of significance, with Bonferroni corrections (

* *α < 0.002) for multiple correlations. c-RST: % corr (z), z-score for children's rhythm synchronization task, percent correct; c-RST: ITI synch (z), z-score for children's rhythm synchronization task, ITI synchrony; TCT: paced (z), z-score for tapping and continuation task, paced tapping variability (baseline);TCT: non-paced (z), z-score for tapping and continuation task, non-paced tapping variability (baseline); c-MDT: Simple (z), z-score for children's melody discrimination task, simple melodies; c-MDT: Transposed (z), z-score for children's melody discrimination task, transposed melodies; c-SSDT (z), z-score for children's syllable sequence discrimination task (baseline)*.

Accounting for multiple correlations, c-RST – percent correct was not significantly correlated with cognitive variables. In contrast, c-RST – ITI synchrony was significantly correlated with both working memory tasks, namely DS [*r*_(130)_ = 0.40, *p* < 0.001] and LNS [*r*_(130)_ = 0.33, *p* < 0.001], but not Matrix Reasoning [*r*_(130)_ = 0.16, *p* = 0.033]. Paced and non-paced tapping were not related to cognitive variables. For the c-MDT, neither Simple nor Transposed Melodies was significantly correlated with cognitive variables. Finally, Syllable Sequences correlated significantly with DS [*r*_(130)_ = 0.33, *p* < 0.001] but no other cognitive variables, when correcting for potentially spurious correlations.

## Discussion

In the present study, we evaluated two tests of musical ability that were developed for school-age children (7–13 years of age), and present z-scores for groups with and without training. Our findings show that the c-RST and c-MDT are acceptably reliable, and that they are sensitive enough to demonstrate differences in performance between children with and without musical training, replicating findings from previous studies using the same tasks in adults. Overall, older children performed better than younger children. However, there were no discernible stepwise increases between age groups. Within-task performance also mirrored adult patterns, with scores decreasing across levels of metrical complexity for the rhythm task and better scores for the Simple compared to the Transposed conditions in the melody task. Using z-scores derived from the untrained sample, we found that music lessons significantly predicted task performance over and above baseline tasks. Finally, we found that, for musically-trained children, performance on rhythm synchronization and syllable sequence discrimination tasks was highly correlated with working memory abilities.

When the c-RST and c-MDT were evaluated for internal consistency, both were found to be adequately reliable. However, reliability for the c-RST was lower than for the c-MDT. This difference likely reflects the smaller number of trials in the c-RST, but may also relate to having selected the “best set” of items on the c-MDT. Researchers using the 40-item c-MDT are therefore strongly encouraged to estimate their own internal-consistency reliability for comparison. We also found that reliability for both tasks was lower for children without musical training. These issues could be addressed by using psychometric techniques based in item response theory. For instance, future iterations of these tasks might include items that adapt to individual differences in ability, such that correct responding leads to more difficult items and vice-versa (Kline, 2011; Harrison et al., 2017). Finally, because these tasks do not assess all aspects of musical skill, we recommend that they be used in combination with other complementary measures previously used with children. For example, rhythm perception ability could be measured with a musical rhythm discrimination task (e.g., Gordon et al., 2015b). Melody production could be measured with a pitch-matching singing task (e.g., Hutchins and Peretz, 2012).

In this child sample, musicians outperformed non-musicians on both musical tasks, consistent with findings from previous studies in adult musicians using the same tasks (Chen et al., 2008; Bailey and Penhune, 2010; Foster and Zatorre, 2010a; Karpati et al., 2016; Matthews et al., 2016). Moreover, the results are consistent with studies comparing children with and without training on other musical tasks (Hyde et al., 2009; Moreno et al., 2009; Roden et al., 2014b; Habibi et al., 2016). We also found the expected within-task effects in our child sample, such that raw scores decreased as task demands increased. For the c-RST, scores were lower as metric regularity (i.e., beat strength) decreased, consistent with previous studies using the RST with adults (Bailey and Penhune, 2010; Matthews et al., 2016). For the c-MDT, all children were better at detecting deviant melodies when presented in the same key rather than a transposed key, which is similar to previous studies with adults (Foster and Zatorre, 2010a,b). As predicted, the oldest children scored highest, the effect of age being strongest on the c-RST. This is supported by a previous finding using the same task (Tryfon et al., 2017), and by more general findings that children's rhythmic abilities improve with age and exposure to the music of their own culture (Trainor and Corrigal, 2010; Stalinski and Schellenberg, 2012). Despite this overall difference, scores did not increase consistently between age groups, especially for the c-MDT. This is similar to a recent large study which found that music perception ability did not increase as a function of age in Brazilian children (Barros et al., 2017). Taken together, this suggests a need to consider non-linear growth trajectories in childhood, such as the monotonic function which has been used to describe the development of musical expertise in adults (Ericsson et al., 1993).

Using z-scores derived from children without musical training, we were able to successfully predict increases in musical task performance from additional years of lessons, over and above the influence of baseline variables (non-paced tapping and phonemic discrimination). For the c-RST, musical training predicted rhythm synchronization ability, over and above the influence of age and baseline variables. However, musicians in our sample do not score as high as adult non-musicians (e.g., Bailey and Penhune, 2012) until they have an average of 5 years of training (see Table 1). The neural substrates of auditory-motor integration develop across childhood, as demonstrated by cross-sectional studies showing that, without musical training, synchronization ability is on par with adult ability by late adolescence (Drake et al., 2000; Drewing et al., 2006; Savion-Lemieux et al., 2009). Thus, it appears that to perform at adult levels on the c-RST children should be at least 14 or have amassed at least 5 years of lessons.

We also found that musically-trained children had less variability in non-paced timing than those without music lessons. This is consistent with adult studies using similar tasks (Repp, 2010; Baer et al., 2015). However, this apparent advantage for musicians appears only at ages 9 and 11 in our sample. This pattern is very similar to a much earlier study in which children with musical experience had lower tapping variability than non-musicians, but only at 8 and 10 years old; there was no difference for the youngest or oldest age groups (Drake et al., 2000). According to Dynamic Attending Theory, the neural oscillations underlying auditory-motor synchronization stabilize as children get older (Drake et al., 2000). These bottom-up timing abilities, which are based in oscillatory entrainment and increase naturally as children get older, may be temporarily enhanced by musical experience in early or middle childhood. This experience-dependent boost in middle childhood may then decline as the underlying mechanisms mature through adolescence, for both musicians and non-musicians. Adult professional musicians, in turn, have the lowest tapping variability as a function of extended practice, the benefits of which extend far beyond the changes due to maturation.

In contrast to rhythm synchronization, musical training was a strong predictor of improvement in melody discrimination ability, for both simple and transposed melodies. Transposition was especially sensitive to musical training, with the highest effect size for additional years of training on task performance. This is consistent with previous research showing that simple discrimination ability stabilizes in childhood (Stalinski and Schellenberg, 2012) whereas, without musical training, development of transposition discrimination is limited, with adolescents and adults performing at close-to-chance levels on this task (Foster and Zatorre, 2010b; Sutherland et al., 2013). Thus, the ability to detect changes in pitch within a transposed model may only develop fully in musically trained individuals. Quite unexpectedly, child musicians performed better on the baseline Syllable Sequence Discrimination Task (c-SSDT) than children without musical training. This is at odds with previous studies with adults where musically trained and untrained participants performed equally (Foster and Zatorre, 2010a; Karpati et al., 2016). On the other hand, it is possible that adults simply process linguistic material more automatically than children, even those with musical training. Thus, children's enhanced performance on the c-SSDT is consistent with a possible transfer effect from music training to language-related skills that is limited to childhood. In addition to enhancing bottom-up (sensory) discrimination thresholds, musical training affects multiple top-down cognitive processes that may contribute to enhancing performance on non-musical tasks, or far-transfer effects (Patel, 2012; Moreno and Bidelman, 2014). One such effect is improved phonological awareness, which is the first stage of learning to read and involves segmenting components of speech as they occur in time (Moreno et al., 2011; Moritz et al., 2013). The c-SSDT requires listening to a pair of syllable sequences and identifying whether one syllable has changed. This may tap into skills related to phonological awareness. Indeed, brief musical training has been found to increase linguistic abilities in young children (Moreno and Besson, 2006; Moreno et al., 2009). Moreover, children at risk of language delays who received 1 year of music lessons showed no decline in basic literacy skills relative to control subjects (Slater et al., 2014).

Finally, we found that musician's z-scores for the c-RST and c-SSDT, but not the c-MDT or TCT, were strongly related to aspects of working memory. Correlations between rhythm synchronization and cognitive performance are consistent with other studies of far-transfer demonstrating a relationship between rhythm and language skills in children. For example, children with specific language impairments score poorly on rhythmic production tasks (Gordon et al., 2016) and tapping variability in adolescents is negatively correlated with reading skill (Tierney A. T. and Kraus, 2013). On the c-MDT we observed an interesting contrast such that, while not statistically significant, Simple melodies related more strongly to Digit Span, whereas Transposed melodies related more to LNS. This is likely because DS requires only immediate auditory memory and attention, whereas LNS requires mental manipulation and thus imposes a heavier demand on working memory and executive control. Although tentative, this may lend additional behavioral evidence to the hypothesis that transposition is distinct from other discrimination abilities (Foster and Zatorre, 2010a; Foster et al., 2013; Sutherland et al., 2013). Moreover, when considered with our regression results, this suggests that transposition relates to higher-order cognitive abilities that are especially sensitive to the impact of musical training in childhood.

## Conclusions

In conclusion, this study demonstrates that we have been successful in developing age-based scores for two reliable and valid tests of musical skill for school-age children that are sensitive to the effects of training. These tasks and the associated z-scores fill an important need for researchers trying to assess the impact of music training in childhood. We hope that they will be important tools for researchers interested in evaluating the impact of musical training in longitudinal studies, those interested in comparing the efficacy of different training methods, and for those assessing the impact of training on non-musical abilities, such as reading skills and other cognitive functions.

## Author contributions

KI was responsible for research design, data collection, data analysis, and writing; AP was responsible for data collection, writing, and editing; NF provided consultation for data analysis, and edited the manuscript; VP provided consultation for research design, data collection and data analysis, and edited the manuscript.

### Conflict of interest statement

The authors declare that the research was conducted in the absence of any commercial or financial relationships that could be construed as a potential conflict of interest. The handling Editor declared a shared affiliation, though no other collaboration, with KI, AP, NF, VP.
