# Supplementary material for: Rhythm and Melody Tasks for School-Aged Children With and Without Musical Training: Age-Equivalent Scores and Reliability
Source: Front Psychol. 2018 Apr 5;9:426. doi: 10.3389/fpsyg.2018.00426 (PMC5895917; doi:10.3389/fpsyg.2018.00426)
Supplement: Supplementary file 1 [file DataSheet1.docx]

Appendix

Table 1

*Raw Score Means and Standard Deviations for 60-item c-MDT and 30-item c-SSDT, by Musicianship and Age Group*

| **MUSICIANS** | **7** | **8** | **9** | **10** | **11** | **13** |
| --- | --- | --- | --- | --- | --- | --- |
|  | **n = 11 (6F)** | **n = 18 (13F)** | **n = 23 (15F)** | **n = 24 (12F)** | **n = 30 (16F)** | **n = 24 (18F)** |
| Simple Melodies | 69.09 (15.92) | 75.56 (10.03) | 76.38 (8.98) | 77.64 (10.88) | 82.56 (7.91) | 76.38 (12.67) |
| Transposed Melodies | 59.39 (9.52) | 57.96 (8.79) | 63.33 (9.74) | 63.75 (13.77) | 65.22 (9.5) | 65.80 (13.07) |
| Syllable Sequences | 71.52 (11.87) | 78.89 (9.63) | 76.96 (8.76) | 75.83 (8.36) | 81.11 (7.5) | 81.74 (10.34) |
| **NON-MUSICIANS** | **7** | **8** | **9** | **10** | **11** | **13** |
|  | **n = 15 (5F)** | **n = 14 (6F)** | **n = 16 (7F)** | **n = 13 (7F)** | **n = 13 (7F)** | **n = 12 (5F)** |
| Simple Melodies | 57.33 (10.63) | 66.43 (12.71) | 61.67 (8.16) | 69.49 (13.93) | 65.90 (7.95) | 75.56 (12.17) |
| Transposed Melodies | 50.89 (6.36) | 52.38 (8.00) | 52.71 (6.11) | 53.33 (9.23) | 52.56 (7.35) | 59.44 (8.27) |
| Syllable Sequences | 69.33 (10.56) | 66.43 (9.10) | 73.54 (9.46) | 80.00 (8.82) | 77.44 (11.72) | 74.44 (11.40) |

*Note*. Outcome is measured as percent correct.
